# Highly Stable and Flexible Pressure Sensors with Modified Multi-Walled Carbon Nanotube/Polymer Composites for Human Monitoring

**DOI:** 10.3390/s18051338

**Published:** 2018-04-26

**Authors:** Yin He, Yue Ming, Wei Li, Yafang Li, Maoqi Wu, Jinzhong Song, Xiaojiu Li, Hao Liu

**Affiliations:** 1 School of Textiles, Tianjin Polytechnic University, Tianjin 300387, China; smileheyin@yeah.net (Y.H.); yming2017@sinano.ac.cn (Y.M.); lweiyuanb@163.com (W.L.); liyafang@tjpu.edu.cn (Y.L.); wwumaoqi@163.com (M.W.); lixiaojiu@tjpu.edu.cn (X.L.); 2 School of Fashion and Art, Tianjin Polytechnic University, Tianjin 300387, China; 3 Institute of Smart Wearable Electronic Textiles, Tianjin Polytechnic University, Tianjin 300387, China; 4 College of Biomedical Engineering & Instrument Science, Zhejiang University, Zhejiang 310027, China; sjinzhongkx@163.com

**Keywords:** modified multi-walled carbon nanotubes, polyurethane composite films, flexible pressure sensors, electrical conductivity, piezoresistive property

## Abstract

A facile method for preparing an easy processing, repeatable and flexible pressure sensor was presented via the synthesis of modified multi-walled carbon nanotubes (m-MWNTs) and polyurethane (PU) films. The surface modification of multi-walled carbon nanotubes (MWNTs) simultaneously used a silane coupling agent (KH550) and sodium dodecyl benzene sulfonate (SDBS) to improve the dispersibility and compatibility of the MWNTs in a polymer matrix. The electrical property and piezoresistive behavior of the m-MWNT/PU composites were compared with raw multi-walled carbon nanotube (raw MWNT)/PU composites. Under linear uniaxial pressure, the m-MWNT/PU composite exhibited 4.282%kPa^−1^ sensitivity within the pressure of 1 kPa. The nonlinear error, hysteresis error and repeatability error of the piezoresistivity of m-MWNT/PU decreased 9%, 16.72% and 54.95% relative to raw MWNT/PU respectively. Therefore, the piezoresistive response of m-MWNT/PU had better stability than that of raw MWNT/PU composites. The m-MWNT/PU sensors could be utilized in wearable devices for body movement detection, monitoring of respiration and pressure detection in garments.

## 1. Introduction

Carbon nanotubes (CNTs) are promising carbon materials for wearable electronic sensor because of their remarkable mechanical and electrical properties with large aspect ratio [1,2,3]. CNT-based pressure sensors can be utilized for e-skin, human healthcare and motion detective devices and smart textiles [2,4,5]. Michelis et al. [6] built a highly reproducible and hysteresis-free flexible strain sensor by inkjet printing carbon nanotubes on ethylene tetrafluoroethylene sheets. Lipomi et al. [7] prepared spray-deposited films of single-walled carbon nanotubes to developed a skin-like sensors which can stretch and bend reversibly or sense touch pressure. Yamada et al. [8] fabricated aligned CNTs films as flexible piezoresistive sensors to detect human motion such as movement, typing, breathing and speech. Roh et al. [9] reported a stretchable, transparent, ultrasensitive and patchable strain sensor made of a novel sandwich-like piezoresistive film of carbon nanotubes to detect human facial motion such as laughing and crying.

CNTs, as conductive fillers, are dispersed into the polymer matrix by melt or solution mixing methods and the resulting piezoresistive conductive composites are able to convert external stress to electrical signals. The electrical conductive of CNT-filled polymeric composites will have a variation caused by two ways after applied stress. One is tunneling resistance changes due to the change of distance between CNTs, another is intrinsic resistance of CNTs changes due to the composites deformation [10]. Wang et al. [11] reported that carbon nanotubes are preferred for high sensitivity in silicon rubber. The composite can be used as a sensitive material for a flexible pressure sensor. Tai et al. [12] prepared conductive and piezoresistive spheres based on single multi-walled carbon nanotube/alginate hydrogel. The hydrogel sensor exhibited a high sensitivity and low detectable limit to monitoring human wrist pulse, detecting throat muscle motion. Han et al. [13] presented a CNT/PDMS sponge with high piezoresistive and good mechanical properties, which can be used to artificial skin to grip sensitive objects. Jung et al. [14] developed porous pressure-sensitive rubbers by mixing multi-walled carbon nanotubes, PDMS and reverse micellar solutions. Compared with traditional sensors, this sensor response with higher sensitivity.

The piezoresistive behavior of these composites includes sensitivity, linearity, hysteresis and repeatability. These are markedly affected by the distribution of carbon nanotubes in polymers and the interfacial interaction between nano fillers and the polymer matrix [15,16]. Therefore, many studies have been devoted to functionalizing carbon nanotubes/polymer composites to achieve a homogenous dispersion of fillers in the polymer [17,18,19]. Hwang et al. [20] reported that the good sensitivity of MWNT/PDMS composites in small pressure range is based on modified MWNTs by using poly(3-hexylthiophene) wrapping method. Benlikaya et al. [19] used H_2_O_2_, HNO_3_ and KMnO_4_ to oxidize carbon nanotubes and fabricated polyurethane composites with higher sensitivity. The composites were prepared by monitoring the elbow joint flexion during physical exercises.

Otherwise, high-quality piezoresistive sensors depend not only on the effective conductive CNT-filler networks but also on the flexible polymer matrix such as polyurethane (PU) [15,21,22], silicone rubber [10,11,23,24], polydimethylsiloxane (PDMS) [20,25], epoxy resin [26] and polyvinylidene fluoride (PVDF) [27]. The flexibility and extensibility of polymer make the sensor respond to strains such as torsion, tension and compression. Polyurethane elastomers are commonly used as the polymer matrix due to their low modulus, high elasticity, easy processing and good flexibility.

In this work, we present a novel method for fabricating a sensitive, reliable and low-cost flexible pressure sensor based on modified multi-walled carbon nanotube (m-MWNT)/polyurethane (PU) composites and polydimethylsiloxane (PDMS) films. The two modifiers including the γ-aminopropyl-triethoxy silane (KH550) and sodium dodecyl benzene sulfonate (SDBS) were employed to decorate the MWNTs. Subsequently, the m-MWNTs were dispersed into polyurethane and *N*,*N*-dimethylformamide (DMF) solution to mold the m-MWNT/PU composite films. The raw MWNT/PU was also prepared under similar conditions to investigate the effects of surface modification of MWNTs on the electrical and piezoresistive properties of the composites. Static and dynamic resistance responses of the composites under compressive loading/unloading were also studied. With the ultrastability of piezoresistivity, m-MWNT/PU and PDMS were fabricated as pressure sensors for use in applications of flexible pressure detection such as human motion sensing, physiological signal detection and so on.

## 2. Experiments

### 2.1. Materials

Multi-walled carbon nanotubes (MWNT-OH) were provided by Chengdu Organic Chemicals Co. Ltd., Chinese Academy of Sciences (Chengdu, China). The outside diameter of MWNT is >50 nm; the length is 10–20 μm; the purity is >98 wt %. Polyurethane resin (PU) was synthesized by means of solution polymerization process with DMF and 4,4′-diphenylmethane diisocyanate, poly ethylene propylene adipate glycol, 1,4-butylene glycol which were provided by China HuaDa Industrial Co., Ltd. (Yantai, China). Anhydrous ethanol which was purchased from Tianjin Kemiou Chemical Reagent Co., Ltd. (Tianjin, China) was used as the solvent to prepare the MWNTs and the composites. The KH550 and SDBS which were purchased from Beijing Chemical Reagent Company were used to treatment MWNTs.

### 2.2. Modification of MWNTs

During the modification process, MWNTs were modified with SDBS as the surfactant. The surface of the MWNTs was simultaneously treated with KH550. The raw MWNTs were added into ethanol at the same mass fraction of SDBS and KH550 (1 wt %). The solution with MWNTs and two modifiers were mixed and ultra-sonicated for 2 h at 80 W at room temperature. The mixed solution was then centrifuged at 6000 rpm for 30 min. The modified MWNTs were collected by drying at 100 °C after vacuum filtration of the suspension.

### 2.3. Preparation of Modified-MWNT/PU Composites

The nanocomposites of the modified MWNTs and polyurethane (m-MWNT/PU) with different contents of m-MWNTs were fabricated via the solution mixing method (Table 1). First, the m-MWNTs were ultrasonically dispersed in DMF solvent for 20 min. Second, PU resin was added into the solution with m-MWNTs to mix followed by magnetic stirring for 2 h. The ultrasonic vibration m-MWNT/PU suspension for 30 min created a better filler dispersion. The suspension then stood in vacuum to degas and avoid trapped bubbles. Finally, the homogeneous solution of m-MWNT/PU was cast in the glass mold and this was dried in a vacuum oven for 3 h at 85 °C. The specification of the samples was 4 × 7 cm^2^ with a thickness of 50 μm. At the same time, the raw MWNT/PU composite films was prepared similarly to compare the properties of the modified samples.

### 2.4. Characterization

A Delza Nano Particle size analyzer (Beckman Counlter, Brea, CA, USA) was used to measure whether the MWNTs agglomerates. Prior to measurement, the raw MWNTs and m-MWNTs were separately added into DMF and ultrasonic dispersed about 30 min. A Transmission Electron Microscope (TEM) (HITACHI 800, Hitachi Ltd., Tokyo, Japan) and a Emission Scanning Electron Microscope (SEM) (HITACHI H-7650, Hitachi Ltd., Tokyo, Japan) were used to observe the morphology of a single of MWNTs and MWNT/PU composite films, respectively.

**Figure 1:**
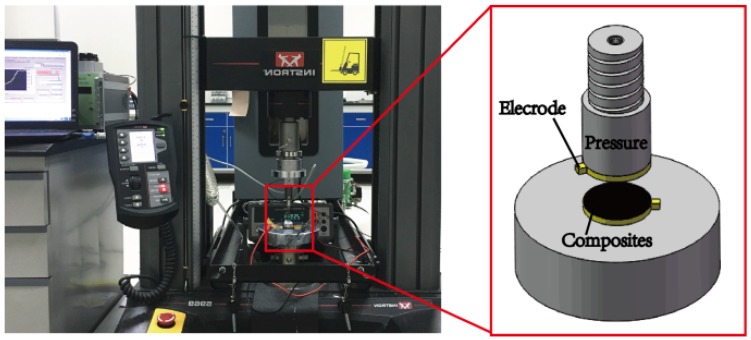
Experimental set up for measurements of piezoresistive m-MWNT/PU and raw MWNT/PU composite films.

A Raman spectrometer (RFS/100s, Bruker Corporation, Karlsruhe, Germany) and Fourier Transform Infrared (FTIR) spectrometer (BRUKER TENSOR 27, Bruker Corporation, Karlsruhe, Germany) characterized MWNTs and MWNT/PU composite films from 400–4000 cm^−1^ of the scanning frequency. Electrical conductivity experiments used a dielectric spectrometer (BOS50, NOVO Control GmbH Co., Montabaur, Germany) at 20 °C. The dielectric permittivity of raw MWNT/PU and m-MWNT/PU composite was measured in the frequency range of excitation signal from 10^−1^ Hz to 10^7^ Hz and the excitation voltage of the alternating current was 10 mV. The pressure-resistivity tests used a universal strength tester (INSTRON 5969, Instron GmbH, Norwood, MA, USA) with a 500.0 N load cell about ±0.5% at load cell capacity of 1/200 to loading and unloading compressive force at different speed rates of 5 mm/min and 10 mm/min and a digital dual display multimeter (U3402A, Agilent, Santa Clara, CA, USA) to monitor the resistance of the samples simultaneously. The composite sample has a diameter of 10 mm and a thickness of about 100 μm and the both sides of the sample was applied a copper sheet, as shown in Figure 1. The real-time piezoresistivity changes of the composites were recorded by LabVIEW 2011 software.

## 3. Results and Discussion

Table 2 shows the diameters of raw MWNTs and m-MWNTs prepared with different modifiers. However, the results of raw MWNTs is 173.5 ± 16.37 nm, which is much larger than the diameter of a single raw MWNT (50 nm). These results illustrate that the aggregates of raw MWNTs occur in the DMF solution before testing. However, the diameter of m-MWNTs used a combination of (KH550 + SDBS) and reaches 62.05 ± 0.45 nm, which is ~36% of raw MWNTs. Meanwhile, the diameter of MWNTs with (KH550 + SDBS)-modified MWNTs is smaller than those with KH550- or SDBS-modified ones. The results agree with Table 2. The modifier can effectively reduce the aggregation of MWNTs.

**Figure 2:**
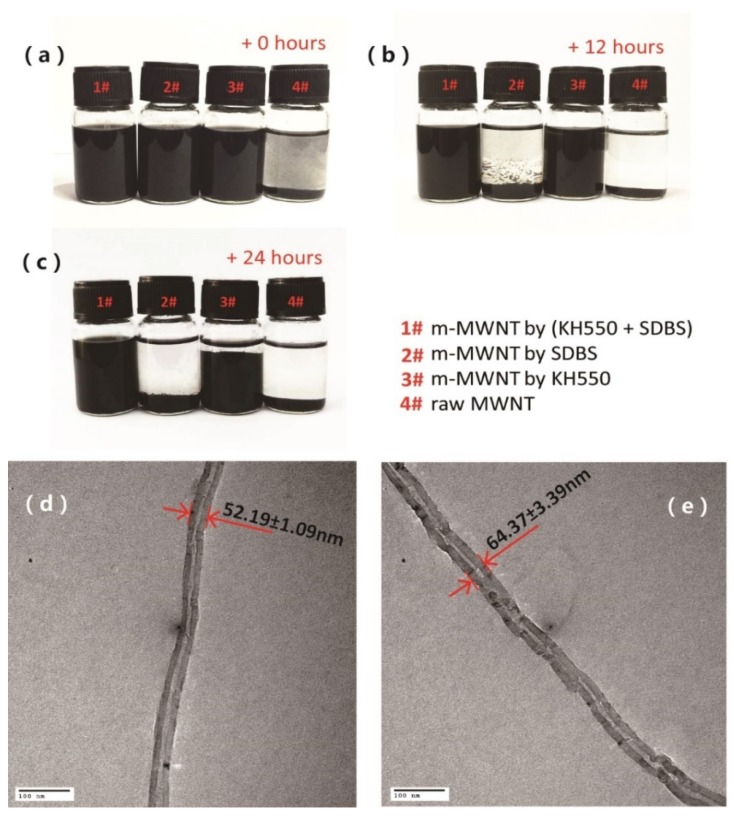
(**a**–**c**) Photographs of MWNTs dispersed in *N*,*N*-dimethylformamide (DMF) and placed for 0 h (**a**), 12 h (**b**), 24 h (**c**). #1 modified MWNTs by using KH550 and sodium dodecyl benzene sulfonate (SDBS); #2 modified MWNTs by using SDBS; #3 modified MWNTs by using KH550; and #4 raw MWNTs. (**d**,**e**) Transmission electron microscopy (TEM) images of raw MWNT (**d**) and m-MWNT by KH550 and SDBS (**e**).

Figure 2a–c shows m-MWNTs and raw MWNTs dispersed in DMF solvent with ultrasonication for 20 min, which were then allowed to stand for 24 h. Figure 2a shows that the modified MWNTs (1#, 2#, 3# reagent bottle) are better dispersed than the raw MWNTs (4# reagent bottle) in DMF. After settling for 12 h, sediments of raw MWNTs (1# reagent bottle) are seen in Figure 2b. The dispersions of the m-MWNTs (1#, 2#, 3# reagent bottle) 24 h later have precipitates in 2# and 3# bottles, as shown in Figure 2c. Thus, the dispersibility of m-MWNTs by using (KH550 + SDBS) in tandem is much better than that of raw MWCNT, which indicated that the surface treatment of MWNTs with concurrent KH550 and SDBS can improve the homogenous and stable distribution of MWNTs into the polymers.

Figure 2d,e shows TEM images of a single raw MWNT and m-MWNT. The raw MWNTs and m-MWNTs (KH550 + SDBS) samples were casted on copper mesh from 0.001 wt % solution in DMF. Figure 2d shows that the raw MWNTs have a comparatively smooth and clean surface. The average diameter of this single raw MWNT and m-MWNT was 52.19 ± 1.09 nm and 64.37 ± 3.39 nm, respectively, after 10 measurements. The diameter of the m-MWNT is larger than that of the raw MWNT indicating that the surface of MWNT was wrapped by a layer of SDBS. Moreover, the rough surface appears on the edges of m-MWNT (KH550 + SDBS) due to the attachment of groups on MWNT by KH550.

**Figure 3:**
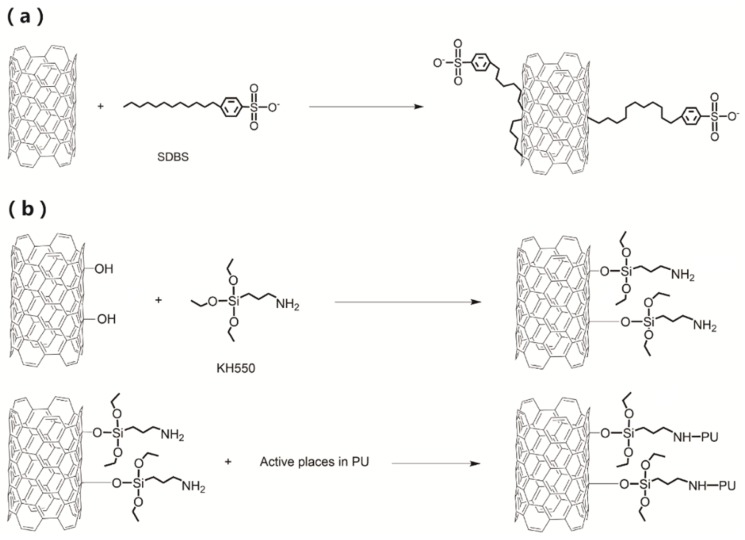
(**a**) Schematic processes of physical adsorption between MWNTs and SDBS; (**b**) chemical interaction of KH550 with MWNTs and PU.

The SDBS are disorderly absorbed onto and squeezed into the MWNTs sidewall with the hydrophobic alkyl chain tail via van der Waals forces and π-like stacking of the benzene rings onto the surface of MWNTs. The sulfonic head group is hydrophilic and is repelled from the MWNTs (Figure 3a) [28]. This physical absorption of MWNTs and SDBS can reduce the aggregation of nanotubes and gives the MWNTs good compatibility with the polymer [29]. Meanwhile, the hydrolysis of the alkoxide groups of KH550 can combine with the oxygen-containing groups of MWNTs and the amine of KH550 forms hydrogen bond with the oxygen in PU (Figure 3b). Therefore, KH550 can lead to better interfacial interactions between MWNTs and PU.

**Figure 4:**
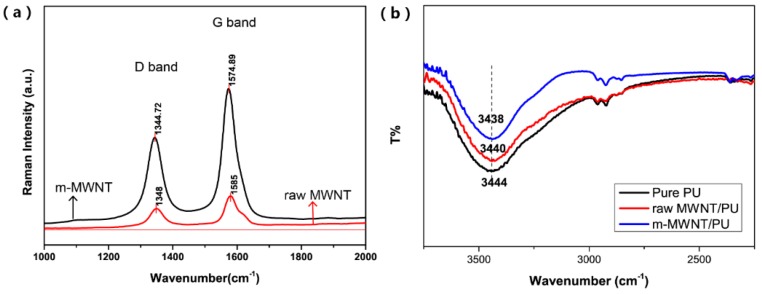
(**a**) Raman spectra of raw MWNTs and m-MWNTs; (**b**) Fourier transform infrared (FTIR) spectra of pure PU, raw MWNT/PU composite and m-MWNT/PU composite.

Figure 4a shows the Raman spectra of raw MWNTs and m-MWNTs to characterize the functionalization of MWNTs using (KH550 + SDBS). Figure 4a shows Raman spectra of raw MWNTs that exhibit a D band at 1348 cm^−1^ and a G band at 1585 cm^−1^; Raman spectra of m-MWNTs have the corresponding bands at 1344.72 cm^−1^ and 1574.89 cm^−1^, respectively. The intensity area ratio of the D to G bands (ID/IG) can evaluate the defect of the surface of MWNTs [18]. Versus the intensity ratio of raw MWNTs (ID/IG = 0.95), the ratio of m-MWNTs increases to 0.97. Considerable defects were produced on the surface of MWNTs with KH550 to graft hydroxyl groups on the sidewalls of MWNTs. However, the increase in ID/IG values in this result is much lower than in the literature [30]. This is because the SDBS wrapped the MWNTs to prevent the hydroxyl groups attached on the MWNTs and this can improve dispersion of MWNTs when the surface defects slightly increase.

Figure 4b illustrates the FTIR spectra of pure PU, raw MWNT/PU composite and m-MWNT/PU composite. The absorption peak at 3444 cm^−1^ was from N–H band stretching and vibrations (free N–H group in the urethane linkage). Figure 4b shows that the N–H stretching peak shifted from 3444 cm^−1^ of pure PU and 3440 cm^−1^ of raw MWNT/PU composites to 3438 cm^−1^ of m-MWNT/PU. The results suggested that m-MWNT/PU is not only MWNTs and PU—There are also strong chemical interactions. The surface of modified MWNTs was linked with more carbonyl groups to provide hydrogen bonding to interact with the N–H bands of PU. Therefore, the functionalized MWNTs can improve the dispersion of nano-fillers in the PU matrix.

**Figure 5:**
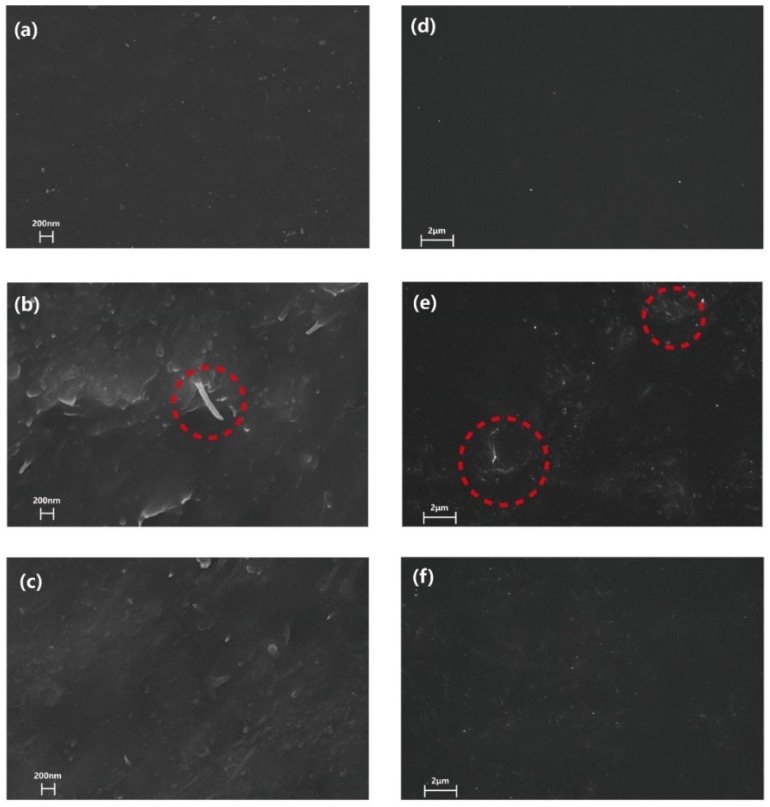
FE-SEM images of cross-sectional fractures of pure PU (**a**), raw MWNT/PU composite, (**b**) and m-MWNT/PU composite (**c**). The surfaces FE-SEM images of the pure PU (**d**), raw MWNT/PU composite (**e**) and m-MWNT/PU composite (**f**).

Figure 5 presents the SEM images of neat PU matrix and raw MWNT/PU composite with 1 wt % fillers content. The m-MWNT/PU composite had 1 wt % filler as well. There is a smooth morphology of pure PU without MWNTs in Figure 5a but there is a single nanotube extraction from the PU matrix. This shows a distinct interface zone between raw MWNTs and PU as seen in the red mark of Figure 5b. The MWNTs modified with the combination of (KH550 + SDBS) were dispersed homogenously and partial extraction of MWNTs in the PU matrix is seen Figure 5c. Figure 5d shows that the surfaces of pure PU are smooth. Regarding 1 wt % raw MWNT/PU composite, rougher surfaces are seen upon adding raw MWNTs in PU. There was obvious agglomeration of raw MWNTs as seen in the red marks of Figure 5e. Meanwhile, well-dispersed m-MWNTs are observed for the 1 wt % m-MWNTs composite (Figure 5f). Therefore, the modification of MWNTs with (KH550 + SDBS) contributes to the better dispersibility and compatibility of the m-MWNTs in PU.

**Figure 6:**
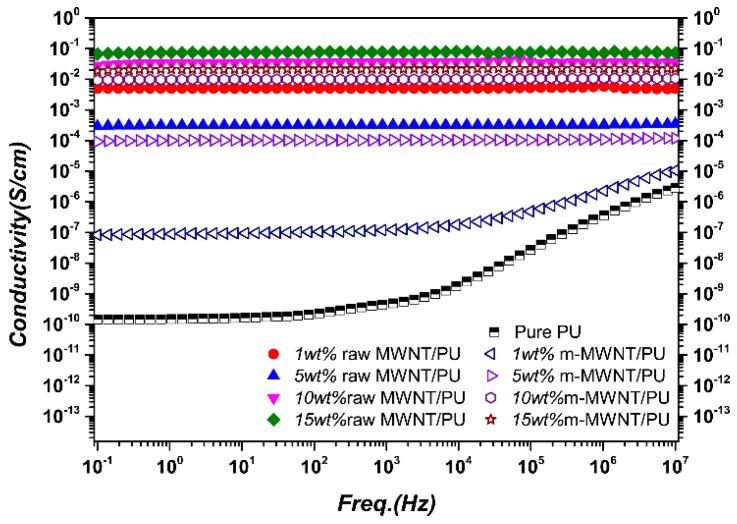
Frequency dependence of electrical conductivity of raw MWNT/PU composite and m-MWNT/PU composite films with varying filler content (wt %).

However, the better interfacial interaction results in poor conductivity of the composites because electron transport is blocked by the energy barrier [29]. Figure 6 shows frequency dependence of conductivities for raw MWNT/PU composites and m-MWNT/PU composites with different filler content fractions. With the MWNTs filler content and frequency increases, the conductivity of the composites increases significantly. PU is an electrically insulating material with measured conductivity values on the order of 10^−11^ S/cm. Figure 6 shows that the addition of MWNTs can be considered as the electrical conductor filled into electrically insulating PU. It then transits them to the conductive composites on the order of 10^−1^ S/cm of 15 wt % raw MWNT/PU.

However, the electrical conductivity of raw MWNT/PU composites is better than the m-MWNT/PU composites regardless of the concentration of the fillers. Although the good dispersion of m-MWNTs was further improved by SDBS to enhance conductivity, the interaction between some MWNTs and the PU chains can cause the appearance of tunneling within the relative PU layers due to the KH550 modifier. The better interfacial interaction produces poor conductivity of the composites because electron transport is blocked by the energy barrier [29]. It is reasonable that the conductivities of m-MWNT/PU composites are not better than the conductivities of the raw MWNT/PU composites.

**Figure 7:**
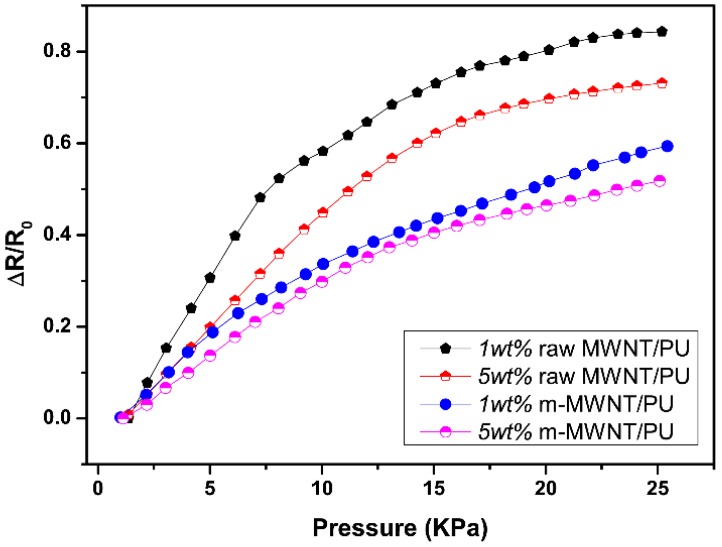
The pressure-resistivity responses of raw MWNT/PU and m-MWNT/PU composite films with different filler contents: 1 wt % and 5 wt % in applied pressure.

Figure 7 shows the positive pressure-sensing capability of the composites in the direction of compression. The relative resistance curve can increase with increasing pressure because the MWNTs effectively reconstruct the conductive paths in the matrix and modify the tunneling distance between MWNTs during the compressive deformation of the composites [31]. The relative resistance curves of raw MWNT/PU composites show become sharp at low pressure. There is then a slow trend in continuous applied pressure but the relative resistance curves of m-MWNT/PU composites keep rising (Figure 7).

The non-linear error of the composites can be calculated as

(1) $$\gamma_{N}=\pm \frac{\Delta L_{\max}}{Y_{\mathrm{FS}}}\times 100\%$$

where $\Delta L_{\max}$ is largest deviation of a real transfer function from the fitting straight line; $Y_{\mathrm{FS}}$ is the measuring range. After linear fitting of the relative resistance for loading compression, the nonlinear errors of 1 wt % and 5 wt % raw MWNT/PU composites are ±17% and ±18%, respectively. The nonlinear errors of 1 wt % and 5 wt % m-MWNT/PU composites are ±8% and ±11%, respectively. The good linear response of piezoresistivity for m-MWNT/PU compared with raw MWNT/PU composites is because the modified MWNT has better dispersion and stronger interfacial action with the PU.

The piezoresistive sensitivity of the composites can be expressed as the following formula [32]:

(2) $$S=\frac{\left(\Delta R/R_{0}\right)\%}{\Delta P}$$

(3) $$\Delta R=\left|R-R_{0}\right|$$

∆P is the relative loaded compressive force, R_0_ is initial resistance and R is the resistance. Table 3 summarizes the sensitivity of the composites under varying applied pressures. From 0–1 kPa, the 1 wt % raw MWNT/PU exhibits the highest sensitivity of piezoresistive effect (8.372%kPa^−1^). However, the sensitivity of 1 wt % m-MWNT/PU is only 4.282%kPa^−1^ at the same pressure range (0–1 kPa). This obvious difference in sensitivity is determined by the electrical conductivities of two composites. Increasing the MWNTs content results in decreasing sensitivity because the increase in MWNTs leads to more aggregation.

**Figure 8:**
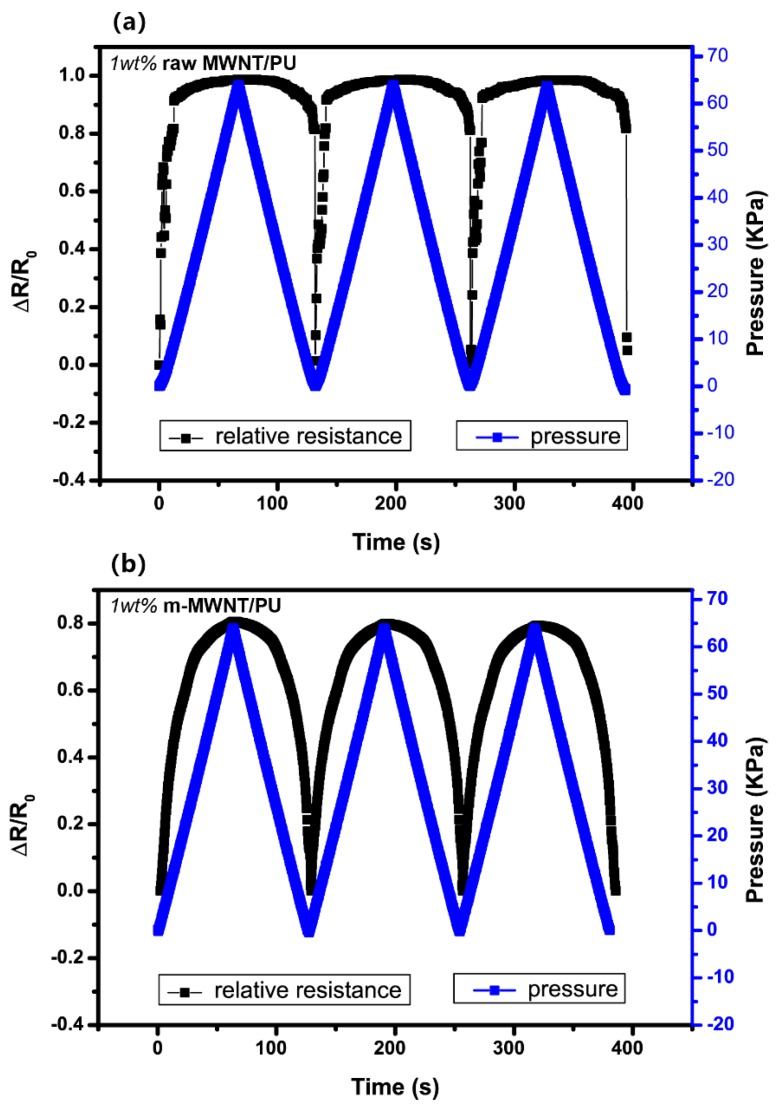
Comparison of piezoresistive responses of the composite with same range of cyclic pressure as a function of time (**a**) 1 wt % raw MWNT/PU (**b**) 1 wt % m-MWNT/PU.

The piezoresistive of raw MWNT/PU and m-MWNT/PU composites exposed to three cycles of 63 kPa pressure loading (compared in Figure 8). All composites exhibit the stability and non-reversibility drift in loading/unloading. Figure 8a shows that the piezoresistive points of raw MWNT/PU composites are unstable at the transition between loading and unloading. Figure 8b shows that the piezoresistive response of m-MWNT/PU has less amplitude than the raw MWNT/PU composite but is exempt of noise. These results clearly indicate that the repeating piezoresistive behavior of m-MWNT/PU composites are attributed to the stable conductive network constructed by a more homogeneously modified MWNTs.

**Figure 9:**
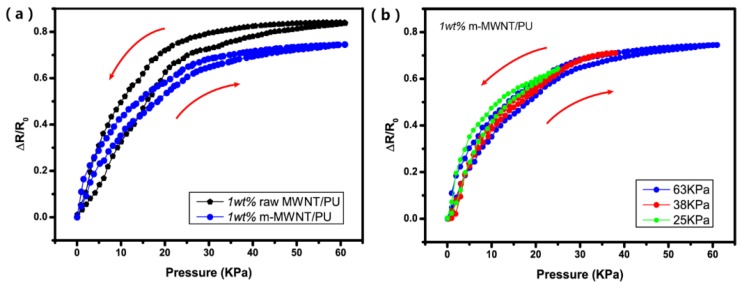
(**a**) A cyclic pressure-resistivity response of raw MWNT/PU composite and m-MWNT/PU composite; (**b**) a cyclic pressure-resistivity response of m-MWNT/PU composite at different applied pressures.

Figure 9 shows the cyclic piezoresistive curves for the raw MWNT/PU and m-MWNT/PU composites versus applied compression. To quantify the correlation of the composites pressure-resistivity, the hysteresis error of the piezoresisitvity can be calculated by:

(4) $$\gamma_{H}=\pm \frac{\Delta H_{\max}}{Y_{\mathrm{FS}}}\times 100\%$$

(5) $$\Delta H_{\max}=\mathrm{MAX}\left|R_{L}\left(s,t\right)-R_{u}\left(s,t\right)\right|$$

Here, $\Delta H_{\max}$ is the largest deviation between the loading and unloading. $R_{L}\left(s,t\right)$ is the resistance of the composite under loading pressure Ps and $R_{u}\left(s,t\right)$ is the resistance of the composite under the unloading pressure Ps.

Figure 9a shows that the hysteresis windows of the curves shift under compression/release cycles. The hysteresis error of the piezoresistivity of the 1 wt % raw MWNT/PU and 1 wt % m-MWNT/PU is ±24.92% and ±8.2%, respectively. The piezoresistive curve of the m-MWNT/PU composite shows a slighter window, which indicates that good recovering performance of the composites are attributed to the high state of MWNTs dispersion throughout the PU matrix.

Moreover, different pressure levels (i.e., 25, 38 and 63 kPa) were applied to the m-MWNT/PU composites and the response of the composite sensors under both compress and release cycle were measured. Figure 9b demonstrates that the resistance of the composite recovered very well after releasing it. A small hysteresis in the response of the composite sensor could be caused by viscoelastic behavior of the PU resin [33].

**Figure 10:**
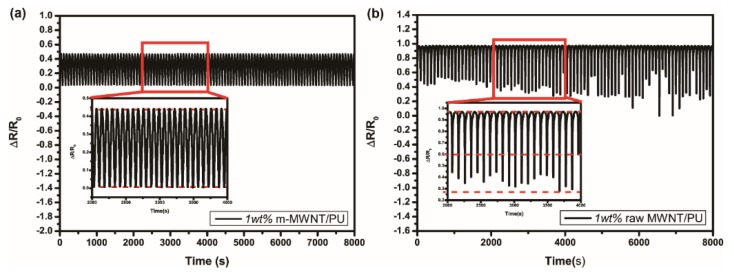
The piezoresistive repeatability of the m-MWNT/PU (**a**) and raw MWNT/PU (**b**) composites were tested by automatic pressing and releasing of 63 kPa for more than 20,000 s; The insets show the cycles for 2000 s.

Figure 10a,b shows that the resistivity decreases with increasing pressure and increases with decreasing pressure at every loading/unloading cycle. Furthermore, the piezoresistive stability of the composites was tested by applied pressure for long time. The repeatability error can be calculated as

(6) $$\gamma_{R}=\pm \frac{\Delta R_{max}}{Y_{FS}}\times 100\%$$

(7) $$\Delta R_{max}=\mathrm{MAX}\left[\Delta R_{Lmax},\Delta R_{Umax}\right]$$

Here, $\Delta R_{Lmax}$ and $\Delta R_{Umax}$ are the maximum deviation of the resistivity among loading and unloading curves. The 1 wt % m-MWNT/PU composite remains nearly constant after repeating pressure. The repeatability error of 1 wt % m-MWNT/PU composite is less than ±6.63%. On the contrary, the relative resistance curve of 1 wt % raw MWNT/PU composite shows a large deviation in the range of change rate. The repeatability error of 1 wt % raw MWNT/PU composite is about ±61.58%. The good dispersion of m-MWNT can construct a stable conductive network to explain the phenomenon precisely.

**Figure 11:**
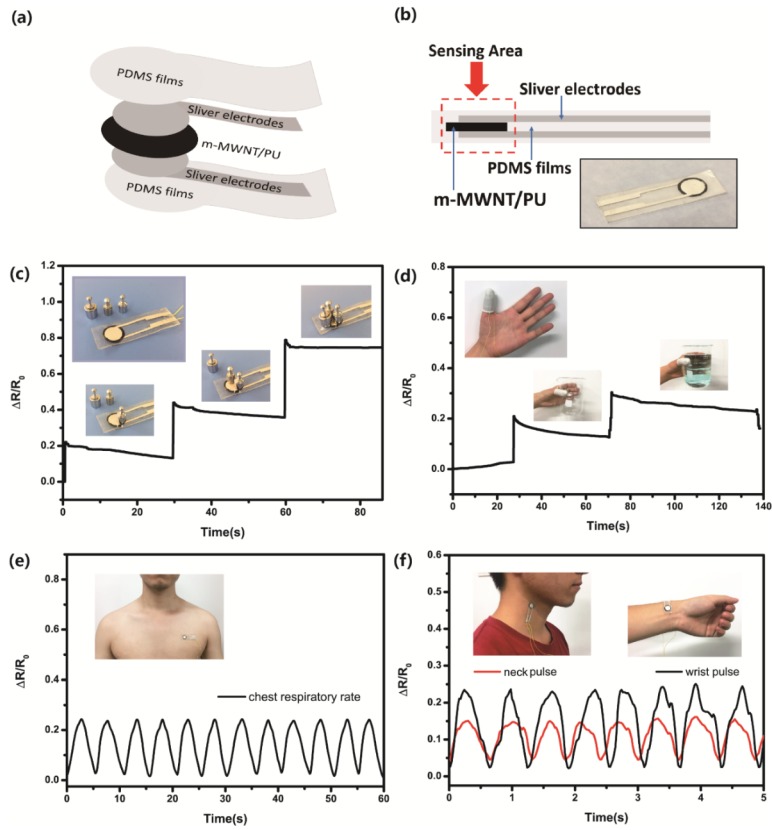
(**a**,**b**) Schematic and photograph of the flexible m-MWNT/PU sensor; (**c**) Detection of the resistance responses to static loading pressure; (**d**) Current response of the pressure sensor under the finger gesture of holding things; (**e**) Relative change of resistance of the human respiratory; (**f**) Relative change of resistance or the human pulse of neck and wrist, respectively.

To illustrate the applicability of the piezoresistivity of m-MWNT/PU composite films, the flexible pressure sensors was fabricated by applying two silver electrodes of 1 wt % m-MWNT/PU films and sealed both sides by PDMS films (Figure 11a,b). Figure 11c–f shows data with the flexible pressure sensors based on 1 wt % m-MWNT/PU.

The m-MWNT sensors can detect and distinguish the static and gentle pressure. Figure 11c show two 1 g standard weights and one 2 g standard weight placed on a sensing area of the sensor one-by-one. The response of the pressure sensor shows weights loading over three times, in which three peaks correspond with smaller relative resistance. In addition, the m-MWNT/PU sensor can be attached onto a human thumb to detect motion (Figure 11d). Once the hand holds an empty beaker, the thumb presses the beaker and the relative resistance increases. The hand then holds a beaker full of water and the relative resistance continues to increase.

The flexible pressure sensor can also be mounted on human bodies for use as a wearable device for health care. Figure 11e shows that the sensor is mounted on the volunteer’s chest to monitor signals of the breathing rate. Each cycle of the piezoresistive cure represents a breath. The respiration frequency range of the volunteer on chest was about 0.2 Hz. The flexible sensor can also detect subtle physiological pulse waves in the neck or wrist (Figure 11f). The sensor can clearly record the repeatable resistive signal of pulse frequency and distinguish the signal of the neck pulse from the wrist pulse. Therefore, the m-MWNT/PU composite sensor to apply in healthcare and human motion monitoring, e-skins, or smart garments.

## 4. Conclusions

In summary, an easy processing, high stable flexible pressure sensor with simple construction was designed and fabricated by mixing and casting of m-MWNT and PU. The experimental results showed that the m-MWNTs can be well-dispersed into the polymer matrix and improve the interaction of the MWNTs with PU by using modifier combination of (KH550 + SDBS). These modifications can obviously reduce the cost of preparation of piezoresistive pressure sensor. For the uniaxial compression test, the sensing capacity of m-MWNT/PU was sensitive and repeatable compared with raw MWNT/PU. The piezoresistive effect of the composites enhances the modification of MWNTs and has resistance response of 4.282%kPa^−1^ sensitivity (±8% nonlinear error), good stability (±8.2% hysteresis error) and extremely recovery performance under pressure range of 0–63 kPa with ±6.63% repeatability error. The nonlinear error, hysteresis error and repeatability error of the piezoresistivity of m-MWNT/PU decreased 9%, 16.72% and 54.95% relative to raw MWNT/PU respectively. These results present that the m-MWNT/PU composites have great feasible as wearable electronic devices for body motion detection, healthcare monitoring and smart textile applications.

## Figures and Tables

**Table 1:** Composition of modified multi-walled carbon nanotube/polyurethane (m-MWNT/PU) composite films.

Sample Name	Polyurethane (g)	Modified MWNTs (g)	Filler Content by Weight (wt %)
*Neat* PU	0.9	-	0
1 wt % m-MWNT/PU	0.9	0.009	1
5 wt % m-MWNT/PU	0.9	0.047	5
10 wt % m-MWNT/PU	0.9	0.100	10
15 wt % m-MWNT/PU	0.9	0.159	15

**Table 2:** Average Diameters of MWNTs (nm).

Types of MWNTs	Raw MWNTs	m-MWNTs by KH550	m-MWNTs by SDBS	m-MWNTs by KH550 + SDBS
Diameters of MWNTs	173.5 ± 16.37	79.5 ± 1.85	113.95 ± 7.06	62.05 ± 0.45

**Table 3:** The piezoresistive sensitivity (Unit: %kPa^−1^) of the raw MWNT/PU and m-MWNT/PU composites over a certain pressure range.

Types of Sensor	Corresponding Pressure			
	0–1 kPa	1–10 kPa	10–15 kPa	15–63 kPa
1 wt % raw MWNT/PU	**8.372**	6.273	3.06	0.604
5 wt % raw MWNT/PU	5.218	5.057	2.758	0.491
1 wt % m-MWNT/PU	**4.282**	3.359	1.88	1.568
5 wt % m-MWNT/PU	3.349	3.3	1.861	1.072
